# Methicillin-resistant *Staphylococcus aureus* Colonization in Veterinary Personnel

**DOI:** 10.3201/eid1212.060231

**Published:** 2006-12

**Authors:** Beth A. Hanselman, Steve A. Kruth, Joyce Rousseau, Donald E. Low, Barbara M. Willey, Allison McGeer, J. Scott Weese

**Affiliations:** * Ontario Veterinary College, University of Guelph, Guelph, Ontario, Canada;; † Mt. Sinai Hospital, Toronto, Ontario, Canada

**Keywords:** Methicillin resistance, Staphylococcus aureus, zoonoses, veterinary, horse, dog, pulsed-field gel electrophoresis, research

## Abstract

Methicillin-resistant *Staphylococcus aureus* (MRSA) was isolated from nares of 27/417 (6.5%) attendees at an international veterinary conference: 23/345 (7.0%) veterinarians, 4/34 (12.0%) technicians, and 0/38 others. Colonization was more common for large-animal (15/96, 15.6%) than small-animal personnel (12/271, 4.4%) or those with no animal patient contact (0/50) (p<0.001). Large-animal practice was the only variable significantly associated with colonization (odds ratio 2.9; 95% confidence interval 1.2–6.6). Pulsed-field gel electrophoresis identified 2 predominant clones with similar distribution among veterinarians as previously reported for horses and companion animals. Canadian epidemic MRSA-2 (CMRSA) was isolated from 11 small-animal and 2 large-animal personnel from the United States (n = 12) and Germany (n = 1). In contrast, CMRSA-5 was isolated exclusively from large-animal personnel (p<0.001) in the United States (n = 10), United Kingdom (n = 2), and Denmark (n = 1). MRSA colonization may be an occupational risk for veterinary professionals.

## Abstract 2

TOC Summary: Prevalence of colonization was 6.5%, and employment within a large-animal practice was a significant risk factor.

Methicillin-resistant Staphylococcus aureus (MRSA) is a problematic pathogen in human medicine and appears to be an emerging problem in veterinary medicine. Historically, hospital-associated MRSA infections have predominated in humans and contributed to significant illness and death (*1**–**4*). Recently, a shift in the epidemiology of MRSA infection has been documented, whereby community-associated (CA)-MRSA infections have become more common (*5**–**9*). CA-MRSA may arise from hospital-origin clones that are carried into the community and then transmitted between persons or from de novo development of resistance through acquisition of resistance factors (mecA) by methicillin-sensitive strains of S. aureus (*10*). Asymptomatic colonization with MRSA represents a major risk factor for infection or for transmission among persons within hospitals or the community (*11*).

While CA-MRSA infections are becoming more widely reported, the prevalence of MRSA carriage overall remains low in healthy persons in the community (*6**,**12**–**14*). Reported prevalence of MRSA colonization in the community has been variable; the study population has a major effect on MRSA carriage rates. In the absence of recognized risk factors, the prevalence of colonization tends to be low. In a 2003 study, Salgado et al. identified MRSA colonization in 1.3% of persons overall but in only 0.2% of persons with no identified healthcare-associated risk factors (*12*). A study from Switzerland reported MRSA colonization in 0.09% of persons at the time of hospital admission (*6*). The prevalence of MRSA carriage was 0.3% in a 2005 study that Nulens et al. conducted at a European conference for physicians and others involved in clinical microbiology and infectious disease (*15*).

MRSA infection and colonization have been reported in horses, dogs, cats, birds, and cattle (*16**–**19*). Transmission of MRSA between animals and humans has been reported (*20**–**23*) as have human MRSA infections from animal contact (*16**,**21**,**24*). Recent studies have identified high colonization rates in humans who have close contact with animals. MRSA colonization of persons who work with horses in Canada and the United States was 13% (14/107); on every farm where MRSA was identified in a horse, at least 1 person was colonized (*25*). In another study, 10 (9.7%) of 103 tested veterinary hospital personnel in a large-animal clinic were colonized with MRSA, and clinical skin infections were reported in 3 (*26*). Isolates from horses and humans in each of these studies were indistinguishable by pulsed-field gel electrophoresis (PFGE) and were typed as Canadian epidemic MRSA (CMRSA)-5 (ST8:MRSA:SCCmecIV, also known as USA500), which suggests transmission between horses and humans (*27*). A study at a small-animal referral hospital in the United Kingdom reported MRSA colonization in 17.9% of veterinary personnel. Investigation of clinical infection in 5 dogs and 3 cats found colonization in 14 (16%) of 88 household contacts or veterinary personnel (*28*). In all of the above reports, a screening bias for MRSA colonization may have been present if an outbreak had been ongoing in the population. Whether these results would accurately reflect the prevalence of MRSA in the general veterinary population, and therefore the occupational risk of MRSA exposure for veterinarians, is unclear. Our objective was to determine the prevalence of MRSA colonization in veterinary personnel attending an international veterinary conference and to characterize recovered MRSA isolates.

## Materials and Methods

### Study Population

This study was performed at the annual American College of Veterinary Internal Medicine Forum held in Baltimore, Maryland, USA, June 3–5, 2005. The conference was attended by 3,240 persons: 2,744 practicing veterinarians, 354 technicians, and 142 other veterinary personnel involved in industry or research. Most (86%) attendees were from the United States; however, 43 other countries were represented. An information and sampling booth attended by the investigators was used to enroll adult volunteers; all attendees were eligible. This study was approved by the University of Guelph Research Ethics Board.

### Sample Collection

Participants provided a single nasal swab sample each, which they collected themselves according to instructions to insert a cotton-tipped swab ≈1 cm into each nostril. The swabs were placed in liquid Stuart medium and maintained at 4°C until processing.

Participants completed a brief questionnaire designed to identify potential risk factors for MRSA colonization: nationality, occupational position, type of clinical practice, veterinary patient contact, known exposure to MRSA in veterinary practice, previous hospitalization (within 30 days), previous MRSA infection, and residence with a healthcare worker. Practice types were small-animal (primarily dogs and cats), large-animal (primarily horses but also ruminants), and mixed (combination of large and small animals). We defined CA-MRSA colonization as MRSA isolation from a person with no history of healthcare-associated risk factors.

### MRSA Identification, Characterization, and Typing

Swabs were placed into 2 mL of enrichment broth consisting of 10 g/L Tryptone T (Oxoid Inc., Nepean, Ontario, Canada), 75 g/L sodium chloride, 10 g/L mannitol, and 2.5 g/L yeast extract and incubated for 24 h at 35°C. Approximately 100 µL of broth was spread onto mannitol-salt agar with 10 g/L cefoxitin and incubated at 35°C for 48 h. Isolates were identified as S. aureus on the basis of colony morphologic features, gram-positive stain, catalase-positive reaction, positive tube coagulase test result, and positive latex agglutination test result (Pastorex Staph Plus, Bio-Rad Laboratories Ltd, Mississauga, Ontario, Canada). Methicillin-resistance was confirmed by demonstration of penicillin binding protein 2a with a latex agglutination antibody screening kit (Denka Seinken Co. Ltd, Tokyo, Japan). Antimicrobial susceptibility was performed by Kirby-Bauer disk diffusion according to the Clinical Laboratory Standards Institute (CLSI) guidelines (*29*); mupirocin MIC was determined by using E-Test gradient strips (AB Biodisk, Solna, Sweden). MRSA isolates were typed by SmaI PFGE and categorized as different CMRSA types as described previously (*8*). Real-time PCR was used to detect the lukF and lukS components of the Panton-Valentine leukocidin (PVL) gene previously described (*30*).

### Statistical Analysis

Categorical comparisons were performed using χ^2^ analysis or Fisher exact test. A p value <0.05 was considered significant for all comparisons. Risk factors for MRSA colonization were evaluated by using stepwise forward logistic regression. Variables achieving a liberal significance level of p<0.20 in the univariate analyses were considered for inclusion in the multivariate model. The presence of confounding was evaluated by noting the effect of elimination on the coefficients of the remaining variables. Variables achieving p<0.05 in the final model were considered significant, and odds ratios (ORs) with 95% confidence intervals (CIs) were calculated.

## Results

Nasal swabs were provided by 417 attendees from 19 countries. MRSA was isolated from the nasal cavities of 27 (6.5%) of 417 persons: 15 (15.6%) of 96 in large-animal practice, 12 (4.4%) of 271 in small-animal practice, and 0 of 50 in industry or research (p<0.001) (Table 1). The prevalence of colonization was higher in men (13/139, 9.4%) than in women (14/265, 5.3%); however, this difference was not statistically significant (p = 0.09).

**Table 1:** Risk factors for MRSA colonization among veterinary conference attendees, Baltimore, Maryland, USA, June 3–5, 2005*

Variable	MRSA-colonized persons, n/N (%)	p value	
In clinical practice		0.01
Yes	27/367 (7.4)	
No	0/47	
Animal type†		<0.01
Large	15/96 (15.6)	
Small	12/271 (4.4)	
Practice/facility type		
Academic	11/136 (8.1)	0.29
Private specialty	10/103 (10)	0.10
General	6/139 (4.3)	0.97
Other	0/36	0.97
Position		0.96
Veterinarian	23/345 (7.0)	
Technician	4/34 (12.0)	
Personally diagnosed MRSA in an animal		0.12
Yes	19/275 (6.9)	
No	7/48 (13)	
MRSA case identified at clinic		0.045
Yes	15/244 (6.2)	
No	12/97 (12)	
Personally had diagnosis of MRSA infection or colonization		0.98
Yes	0/4	
No	27/363 (7.4)	
Hospitalized within past 30 d		0.28
Yes	1/5 (20)	
No	26/362 (7.2)	
Healthcare worker in household		0.81
Yes	2/33 (6.1)	
No	25/309 (7.5)	

*MRSA, methicillin-resistant *Staphylococcus aureus*. †Clinical practice only.

Colonized persons were from the United States (n = 23), United Kingdom (n = 2), Denmark (n = 1), and Germany (n = 1). Difference in the prevalence of colonization between countries was not significant (p = 0.18). Colonization rates in persons from the United States, Canada, and the United Kingdom—the 3 countries with the highest representation—were 6.0%, 0%, and 17%, respectively (p = 0.11). According to stepwise forward logistic regression, employment in a large-animal practice was the only variable independently associated with MRSA colonization (OR 2.9; 95% CI 1.2–6.6).

Characterization of MRSA isolates, using PFGE, identified 2 predominant clones: CMRSA-5 (ST8-MRSA-IV, similar to USA500) and CMRSA-2 (ST5-MRSA-II, similar to USA100) (*8**,**31*). CMRSA-5 was isolated from 13 (48%) of 27 colonized persons, all of whom were in large-animal practice. These persons were from the United States (n = 10), United Kingdom (n = 2), and Denmark (n = 1). CMRSA-2 was isolated from 13 (48%) of 27 colonized persons: 11 in small-animal practice and 2 in large-animal practice from the United States (n = 12) and Germany (n = 1). One other isolate, possibly related to CMRSA-2, was recovered from a US veterinarian in small-animal practice. Overall, CMRSA-5 was more commonly isolated from persons in large- than in small-animal practice (p<0.001). A cluster of 5 colonized persons was identified from a US school of veterinary medicine, where 4 persons who worked with large animals were colonized with CMRSA-5, and 1 veterinarian who worked with small animals was colonized with CMRSA-2. No isolates were identified as carrying the lukF or lukS genes. All isolates were susceptible to vancomycin; other antimicrobial drug susceptibility results are shown in Table 2.

**Table 2:** Antimicrobial susceptibility of MRSA isolates recovered from veterinary conference attendees, Baltimore, Maryland, USA, June 3–5, 2005*

PFGE type	No.	Susceptible isolates, n (%)


Mupirocin	Erythromycin	Ciprofloxacin	Tetracycline	Gentamicin	Rifampin	TMP/SMX
CMRSA-2	13	13 (100)	1 (7.7)	2 (15)	13 (100)	12 (92)	13 (100)	13 (100)
CMRSA-5	13	13 (100)	6 (46)	12 (92)	2 (15)	3 (23)	8 (62)	3 (23)
Other	1	0	0	0	1 (100)	1 (100)	1 (100)	1 (100)
Total	27	26 (96)	7 (26)	14 (52)	16 (59)	16 (59)	22 (81)	17 (63)

*MRSA, methicillin-resistant *Staphylococcus aureus*; PFGE, pulsed-field gel electrophoresis; TMP/SMX, trimethoprim/sulfamethoxazole; CMRSA, Canadian epidemic MRSA.

## Discussion

This study represents the most comprehensive evaluation to date of MRSA colonization in veterinary personnel. Although a control group was not included, the prevalence in veterinary personnel (6.5%) was higher than previously reported rates for community-based colonization (*6**,**12**–**14**,**32*). When compared with results from a similar study in which only 0.3% of medical professionals at a conference were colonized (*15*), our results suggest an increased risk for veterinary professionals. However, geographic location and culture methods may have affected the differences in study results, and further investigation is required to more accurately define the occupational risk.

The PFGE type distribution provides support that MRSA is being transmitted between animals and humans. A significant difference was identified between clones recovered from those who worked with large animals (CMRSA-5) and those who worked with small animals (CMRSA-2). If these results merely represented the background level of CA-MRSA colonization in the general population, this difference would not be expected. In addition, CMRSA-5 has not been commonly identified in persons in the community, at least not in Canada (*8**,**33*), although it has accounted for most of the reported cases of MRSA infection or colonization in horses (*16**,**24**,**27*). These findings, along with the identification of CMRSA-5 in large-animal veterinarians from the United States, United Kingdom, and Denmark, provide further evidence that CMRSA-5 is widely disseminated in the horse population and may be transmitted between horses and humans. The CMRSA-5 isolate from the Danish veterinarian was further classified as sequence type 8 and Ridom spa type t064, which has been identified in humans in Denmark, Norway, Germany, Belgium, and Sweden (R. Skov, pers. comm.). CMRSA-5 predominance in large-animal veterinarians may infer an occupational health concern for veterinary professionals who have contact with equine patients. Furthermore, this study's finding of large-animal practice as the only variable associated with colonization and the striking prevalence of MRSA colonization in persons who work with large animals (15.6%) also support an occupational risk for MRSA exposure in large-animal practice. Because zoonotic infections have been associated with exposure to CMRSA-5 (*26*), further evaluation of interspecies transmission within large-animal veterinary practices by routine screening of patients and personnel, along with implementation of infection control practices, may be warranted.

Identification of CMRSA-2 primarily in small-animal veterinarians was consistent with previous reports that MRSA isolates from dogs and cats reflect the predominant human strains in the community (*17**,**22*). In Canada, CMRSA-2 is a hospital-origin clone that has emerged in the community as a major cause of CA-MRSA infections in people (*33**,**34*). The lower prevalence of colonization in persons who work with small animals and the predominance of a common community clone make it more difficult to implicate animal contact as the source of MRSA. However, because colonization in persons who work with small animals was higher than community prevalence rates and because MRSA transmission from household pets to humans has been reported (*20**,**21*), dogs and cats must be considered as possible sources of MRSA. A comprehensive evaluation of MRSA colonization in small animals and risk factors for interspecies transmission are required to determine the true occupational risk for MRSA colonization and infection in small-animal veterinary personnel (*17**,**18*).

Although CA-MRSA isolates that express the PVL genes are frequently a cause of severe skin and soft tissue infections in the community (*8**,**9**,**35**–**37*), this virulence factor was not identified in isolates from veterinary personnel. These results are similar to those of previous studies in which MRSA isolates from veterinary species or personnel have been negative for PVL genes (*24**,**26**,**28**,**38*). However, considering the recent dissemination of the PVL-positive USA300 clone throughout North America and that MRSA isolates in dogs and cats often reflect the predominant community clones, PVL-positive clones may emerge in small animals (*36*). Further monitoring of the dynamic epidemiology of CA-MRSA is required to determine whether animals will have any role in dissemination of this clone.

Previous contact with MRSA in a colonized or infected animal was reported by 6.9% of colonized persons but was not significantly associated with MRSA colonization in veterinary personnel. The number of personnel reporting previous contact with an infected animal was low, thereby limiting the ability of this study to identify an association. Intuitively, one would assume that contact with MRSA-infected animals in veterinary practice would represent a high-risk situation; further investigation is required to more accurately determine this risk.

This study has several limitations. First, the sample used was a convenience sample of attendees at the Forum, which allows potential sample bias. Second, a greater proportion of veterinary personnel sampled were in specialty practice, leading to results that, because of different patient populations, may not apply to general practitioners. Third, because this was a cross-sectional study, causality between risk factors and colonization could not be determined; only an association between variables and colonization could be implied. The small sample size from several countries also limited the statistical power to identify an association in these populations. Lastly, because attendees collected their own nasal swabs, sampling variation may have led to underestimation of the prevalence of colonization. The variable sensitivity (75%–93%) of using nasal swab screening alone (*39*) could also have led to false-negative results. Ideally, a randomized sample of general and specialty practitioners would be performed using >1 sampling site to further characterize the prevalence of MRSA colonization in veterinary personnel.

As MRSA expands into the community, changes in its epidemiology are inevitable. The lives of humans and animals, and their microflora, are closely intertwined. MRSA is now a pathogen of domestic animals that can be transmitted between animals and humans. Accordingly, further scrutiny of the roles of animals in MRSA infection and colonization is required. While occupational and recreational exposure to horses may be a risk factor for MRSA colonization, the effect of routine contact with household pets on the global epidemiology of MRSA is still unknown.
